# Challenging Behavior and Related Factors in People with Intellectual Disability Living in Residential Care Centers in Israel

**DOI:** 10.3389/fpubh.2013.00013

**Published:** 2013-05-21

**Authors:** Amanda Sinai, Ariel Tenenbaum, Shoshana Aspler, Meir Lotan, Mohammed Morad, Joav Merrick

**Affiliations:** ^1^National Institute of Child Health and Human Development, Office of the Medical Director, Health Services, Division for Intellectual and Developmental Disabilities, Ministry of Social Affairs and Social ServicesJerusalem, Israel; ^2^Division of Pediatrics, Hadassah Hebrew University Medical Center, Mount Scopus CampusJerusalem, Israel; ^3^Department of Physical Therapy, Ariel University of SamariaAriel, Israel; ^4^Yaski Community Medical Center, Ben-Gurion University of the Negev and Clalit Health ServicesBeer-Sheva, Israel; ^5^Kentucky Children’s Hospital, University of KentuckyLexington, KY, USA

**Keywords:** intellectual disability, Israel, psychotropic medication, mental health, challenging behavior, residential care

## Abstract

**Introduction:** Adults with intellectual disabilities have higher rates of mental ill-health and problem behaviors than the general population.

**Method:** In this study, we present data on trends in challenging behavior in residential care centers in Israel from 1998 to 2008 and further data on trends in employment of psychiatrists from 1998 to 2009 and psychotropic medication use from 1998 to 2008. Data was collected from annual questionnaires sent out to all residential care centers in Israel, from the Office of the Medical Director, Division for Intellectual and Developmental Disabilities, Ministry of Social Affairs and Social Services.

**Results:** Rates of challenging behaviors in people with intellectual disabilities living in residential care centers in Israel continues to rise. Alongside this, trends in regular psychotropic medication use also continues to increase.

**Conclusion:** Consideration of biological, psychological, social, and environmental factors in the assessment and management of people with intellectual disabilities and challenging behaviors is important. This is best conducted using a multidisciplinary approach, which may include psychiatric assessment. Non-pharmacological interventions should always be considered either alongside, or instead of medication.

## Introduction

In Israel, support services for people with intellectual disability are provided by the Ministry of Social Affairs and Social Services. Approximately 10,000 people out of a total of 35,000 registered people with intellectual disabilities in Israel live in residential care (1). Residential or supported care accommodation can vary from larger centers, which house up to 300 residents, to smaller, community based accommodation [in 2009 mean 112.17 (range 21–324) per residential care center].

It is well known that adults with intellectual disabilities have higher rates of mental ill-health and problem behaviors (2). It is therefore important that this population receive appropriate mental health care, although each individual may need adjustments to the standard treatment plan in order to tailor treatment according to their needs. Although people with intellectual disabilities living in residential centers in Israel are entitled to use general mental heath services, in reality this is difficult to access and residential centers often choose to employ psychiatrists on a private basis to review the mental health of their residents.

Challenging behavior can be defined as behavior “of such an intensity, frequency, or duration as to threaten the quality of life and/or the physical safety of the individual or others and is likely to lead to responses that are restrictive, aversive, or result in exclusion” (3). Behaviors that could be described as challenging can include shouting, self injurious behavior, and verbal and physical aggression directed at others. These behaviors may influence the decision regarding accommodation for an individual with intellectual disabilities and may therefore play an important role in determining whether a person lives in residential care.

Challenging behavior is not a diagnosis in itself, and can be due to a number of different reasons, including environmental, social or psychological factors, sensory impairments, communication difficulties, and/or physical or mental health problems. Amongst other professionals, psychiatrists are often asked to assess people with challenging behavior and can play an important role in assessment and management. Psychotropic medications are not generally recommended to be routinely used for challenging behavior in people with intellectual disabilities (unless the cause is related to mental illness) but are sometimes considered on a case-by-case basis. Psychotropic medications have a number of side effects, and the risks and benefits of prescribing these medications should be carefully considered. Tyrer et al. (4) found that although aggression in people with intellectual disability decreased over 4 weeks when participants were given either haloperidol, risperidone, or placebo, it was the group on placebo who showed the greatest reduction in aggression. There is some evidence for the use of risperidone for the management of challenging behavior in children with intellectual disabilities with or without autism (5). In practice, a number of people with intellectual disabilities and challenging behavior remain on psychotropic medication, due to a variety of reasons, including worsening of challenging behavior when medication is reduced. There is a need for further research in this field, as lack of evidence does not necessarily mean that there is evidence that medication use is ineffective (6).

Although trends in physical and mental health problems in people with intellectual disabilities living in residential care centers in Israel have been reported, including data on medication use (7) and employment of psychiatrists (8), as far as the authors are aware, trends in challenging behavior of people with intellectual disabilities living in residential care centers in Israel have not been previously reported in the literature.

## Materials and Methods

In this study, we present data on trends in challenging behavior in residential care centers in Israel from 1998 to 2008 and further data on trends in employment of psychiatrists from 1998 to 2009 and psychotropic medication use from 1998 to 2008.

Data regarding trends in challenging behavior, the employment of psychiatrists, and psychotropic medication use in people with intellectual disability living in residential care in Israel were examined in this study. Data was collected from annual questionnaires (9) sent out to all residential care centers in Israel, from the Office of the Medical Director, Division for Intellectual and Developmental Disabilities, Ministry of Social Affairs and Social Services.

This questionnaire collects information on health and health-related factors of residents living in residential care centers, including demographic information such as age, gender, and level of intellectual disability of the residents. Amongst other things, it also collects information on mental health factors including number of residents with challenging behavior, number of residents on medication (including psychotropic medication), and whether a psychiatrist is employed by the residential care center.

Questionnaires were completed by staff at the residential care centers. Data was given as total numbers from each residential care center and therefore individual residents were anonymized. Residential care centers were anonymized before data entry.

Data from the questionnaire responses was entered on a database and was then compiled into tables. As data is grouped and anonymous, we did not analyze any associations between the trends.

## Results

Table 1 shows the trends in the number of cases of challenging behavior for people in residential care during 1998–2008 together with the total number of residents for each year, the number of people getting chronic medication every day, number of people getting anti-epileptic treatment, psychotropic medication, and the total number of emergency or SOS medication for aggressive behavior.

**Table 1:** **Trends in challenging behavior and medication for people with intellectual disability**.

Year	Total residents (number of centers)	Challenging behavior (%)	Number of residents receiving long-term medication (%)	Number of residents receiving anti-epileptic medication (%)	Number of residents receiving psychotropic medication (%)	Total numbers of SOS (as required) medications given
1998	6,022 (53)	1,599 (26.55)	3,922 (65.13)	1,730 (28.73)	2,741 (45.52)	1,573
1999	6,122 (53)	1,774 (28.98)	4,330 (71.44)	1,816 (29.66)	2,929 (47.84)	5,343
2000	6,213 (53)	1,959 (31.53)	4,785 (77.02)	1,835 (29.53)	2,848 (45.84)	4,735
2001	6,370 (54)	2,184 (34.29)	4,976 (78.12)	1,873 (29.40)	3,240 (50.86)	6,128
2002	6,352 (57)	2,250 (35.42)	5,098 (80.26)	1,967 (30.97)	3,225 (50.77)	6,814
2003	6,500 (57)	2,250 (34.62)	5,233 (80.51)	2,016 (31.02)	3,162 (48.65)	5,632
2004	6,610 (58)	2,487 (37.62)	5,483 (82.95)	2,127 (32.18)	3,473 (52.54)	6,474
2005	6,749 (58)	2,344 (34.73)	5,548 (82.20)	2,043 (30.27)	3,440 (50.97)	6,739
2006	6,840 (59)	2,488 (36.37)	5,890 (86.11)	2,287 (33.44)	3,515 (51.39)	6,297
2007	6,872 (59)	2,729 (39.71)	5,886 (85.65)	2,306 (33.56)	3,515 (51.15)	5,864
2008	6,988 (63)	2,906 (41.59)	6,082 (87.03)	2,295 (32.84)	3,828 (54.78)	6,355

Table 2 shows the number of residential care centers that employed a consultant psychiatrist over the 1998–2008 time span. The amount of hours employed is also translated into equivalent full time positions.

**Table 2:** **Trends in the number of psychiatrists employed by residential care centers in Israel for people with intellectual disability**.

Year	Total residents (number of centers)	Number of centers with psychiatrists	Rate per 1,000 pop	Percent	Equivalent to full time positions	Rate per 1,000 pop
1998	6,022 (53)	16	2.66	30.19	6.49	1.08
1999	6,122 (53)	44	7.19	83.02	11.96	1.95
2000	6,213 (53)	42	6.76	79.25	11.55	1.86
2001	6,370 (54)	44	6.91	81.48	11.77	1.85
2002	6,352 (57)	45	7.08	78.95	14.05	2.21
2003	6,500 (57)	47	7.23	82.46	14.48	2.23
2004	6,610 (58)	50	7.56	86.21	17.53	2.65
2005	6,749 (58)	52	7.70	89.66	18.83	2.79
2006	6,840 (59)	53	7.75	89.83	18.07	2.64
2007	6,872 (59)	51	7.42	86.44	18.10	2.63
2008	6,988 (63)	53	7.58	84.13	20.89	2.99
2009	7,067 (63)	56	7.92	88.89	23.96	3.39

## Discussion

As shown in Table 1, the number of people with challenging behavior living in residential care centers in Israel has increased from just under 27% in 1998 to just under 42% in 2008. We do not have any further information on the age of residents, type of behavior, cause, or management. These results also show an increase in long-term medication use in people with intellectual disabilities living in residential care centers. They also show a general increase in the use of regular psychotropic and anti-epileptic medication. The use of SOS medication has fluctuated through the years, whereas the levels of challenging behavior reported continue to rise.

This increase in challenging behavior may be explained as it may be that residential care centers are supporting a higher proportion of people with challenging behaviors, and those without challenging behaviors are more likely to be living in the community. Alternatively, it may be that residential homes have started identifying behaviors as challenging, whereas, perhaps previously, they were not recognized as challenging behavior. It is not possible to confirm this without further information.

One interesting finding is that over a similar period of time, the proportion of people with intellectual disability living in residential centers taking regular psychotropic medication has increased. This may be linked to the increase in challenging behaviors in this population, although we do not have information on reasons for prescribing and therefore cannot identify the percentage of people on psychotropic medication specifically for challenging behaviors.

Alongside the increase of psychotropic medication use, there was also a general increase in the number of psychiatrists employed by the residential centers for consultation from 1998 to 2002. Although our study did not directly look at the link between psychiatrists and psychotropic medication use, this may have had an impact on psychotropic medication use, as an increase in psychiatrists is likely to lead to an increase in consultations and subsequently may lead to an increase in prescribing of psychotropic medications. These figures may therefore imply that more psychiatrists are reviewing residents in residential care and are subsequently prescribing psychotropic medication for more people with intellectual disabilities.

Interestingly, previous research has shown that, amongst other factors, challenging behavior and review by a consultant psychiatrist is associated with the use of neuroleptic (antipsychotic) medication in adults with intellectual disability (10). Also, people living in residential campus accommodation in the United Kingdom were found to be significantly more likely to receive regular antipsychotic, antidepressant, and/or anti-epileptic medication as well as required (SOS) antipsychotic medication when compared to those living in village communities or dispersed housing (11).

Assessment and management of challenging behavior should consider underlying causes such as physical and mental health problems, sensory and communication issues, and social, environmental, and psychological factors, amongst other things. The person with intellectual disability should be at the center of their management plan. Non-pharmacological interventions, including psychological and behavioral interventions, should always be considered either alongside, or instead of medication (6).

Although psychiatrists are often involved in the assessment and management of people with challenging behavior, it is important to remember that there may be a number of factors contributing to challenging behavior, which, alongside mental health problems, may include physical health problems or environmental factors. The same behavior may pose a challenge for services in one particular environment, but may not be a challenge in another environment.

The Royal College of Psychiatrists, British Psychological Society, and Royal College of Speech and Language Therapists guidelines provide a very helpful framework for assessing and managing challenging behavior in people with intellectual disability and highlight the importance of considering the individual, their environment, and the interaction between the two (3).

Medication and its side effects should be discussed with the individual, and their family and support workers and medications should be regularly reviewed, and reduced if no benefit is found, or if side effects outweigh the benefits. As people with intellectual disabilities may be more sensitive to medications, it is recommended that if psychotropic medications are started, they are started at low doses and titrated up slowly, monitoring for side effects. Residential centers should have measures in place to monitor medication use and side effects, including SOS (as required) medication. The World Psychiatric Association have published a section report on prescribing psychotropic medications for problem behaviors in adults with intellectual disabilities (12).

Further research into challenging behavior in people with intellectual disabilities living in Israel, including assessment, management, type of behavior, and type of environment is required to develop a further understanding of how best to manage this complex area.

## Conclusion

Rates of challenging behaviors in people with intellectual disabilities living in residential care centers in Israel continues to rise. Alongside this, trends in regular psychotropic medication use also continues to increase.

Consideration of biological, psychological, social, and environmental factors in the assessment and management of people with challenging behaviors is important. This is best conducted using a multidisciplinary approach, which may include psychiatric assessment. Non-pharmacological interventions should always be considered either alongside, or instead of medication (6).

## Conflict of Interest Statement

The authors declare that the research was conducted in the absence of any commercial or financial relationships that could be construed as a potential conflict of interest.
